# The SMAC mimetic LCL-161 selectively targets JAK2^V617F^ mutant cells

**DOI:** 10.1186/s40164-019-0157-6

**Published:** 2020-01-02

**Authors:** Brianna M. Craver, Thanh Kim Nguyen, Jenny Nguyen, Hellen Nguyen, Christy Huynh, Sarah J. Morse, Angela G. Fleischman

**Affiliations:** 1 Department of Biological Chemistry, University of California, Irvine, CA USA; 2 Division of Hematology/Oncology, Department of Medicine, University of California, 839 Health Sciences Road, Irvine, CA 92697 USA; 3 Chao Family Comprehensive Cancer Center, University of California, Irvine, CA USA

**Keywords:** Myeloproliferative neoplasm, SMAC mimetic, TNFα

## Abstract

**Background:**

Evasion from programmed cell death is a hallmark of cancer and can be achieved in cancer cells by overexpression of inhibitor of apoptosis proteins (IAPs). Second mitochondria-derived activator of caspases (SMAC) directly bind to IAPs and promote apoptosis; thus, SMAC mimetics have been investigated in a variety of cancer types. particularly in diseases with high inflammation and NFĸB activation. Given that elevated TNFα levels and NFĸB activation is a characteristic feature of myeloproliferative neoplasms (MPN), we investigated the effect of the SMAC mimetic LCL-161 on MPN cell survival in vitro and disease development in vivo.

**Methods:**

To investigate the effect of the SMAC mimetic LCL-161 in vitro, we utilized murine and human cell lines to perform cell viability assays as well as primary bone marrow from mice or humans with JAK2^V617F^–driven MPN to interrogate myeloid colony formation. To elucidate the effect of the SMAC mimetic LCL-161 in vivo, we treated a JAK2^V617F^–driven mouse model of MPN with LCL-161 then assessed blood counts, splenomegaly, and myelofibrosis.

**Results:**

We found that JAK2^V617F^-mutated cells are hypersensitive to the SMAC mimetic LCL-161 in the absence of exogenous TNFα. JAK2 kinase activity and NFĸB activation is required for JAK2^V617F^-mediated sensitivity to LCL-161, as JAK or NFĸB inhibitors diminished the differential sensitivity of JAK2^V617F^ mutant cells to IAP inhibition. Finally, LCL-161 reduces splenomegaly and may reduce fibrosis in a mouse model of JAK2^V617F^-driven MPN.

**Conclusion:**

LCL-161 may be therapeutically useful in MPN, in particular when exogenous TNFα signaling is blocked. NFĸB activation is a characteristic feature of JAK2^V617F^ mutant cells and this sensitizes them to SMAC mimetic induced killing even in the absence of TNFα. However, when exogenous TNFα is added, NFĸB is activated in both mutant and wild-type cells, abolishing the differential sensitivity. Moreover, JAK kinase activity is required for the differential sensitivity of JAK2^V617F^ mutant cells, suggesting that the addition of JAK2 inhibitors to SMAC mimetics would detract from the ability of SMAC mimetics to selectively target JAK2^V617F^ mutant cells. Instead, combination therapy with other agents that reduce inflammatory cytokines but preserve JAK2 signaling in mutant cells may be a more beneficial combination therapy in MPN.

## Background

Evasion of programmed cell death is a common survival technique utilized by cancer cells and one way these cells can evade apoptosis is by upregulating anti-apoptotic proteins such as inhibitor of apoptosis proteins (IAPs) [1]. IAPs inhibit apoptosis by directly binding to and inhibiting pro-apoptotic caspases. However, when a cell is triggered to undergo apoptosis, second mitochondria-derived activator of caspases (SMAC) is released into the cytosol and bind IAP proteins, leading to their degradation. Degradation of IAP proteins induces apoptosis by freeing pro-apoptotic caspases and allowing them to induce cell death. Small molecules which mimic the antagonistic effects of SMAC and lead to degradation of IAP proteins, termed SMAC mimetics, are actively being investigated in both hematologic malignancies and solid tumors [2, 3]. In preclinical studies, SMAC mimetics induce apoptosis in cancer cells directly or indirectly by priming these cells for killing by other cytotoxic agents. SMAC mimetics sensitize cells to TNFα-induced cell death [4]. TNFα is a master regulator of inflammation and induces NFĸB activation among other signaling pathways. However, NFĸB activation is critical for SMAC mimetic-induced apoptosis [5]. In a study involving primary AML samples, sensitivity to a SMAC mimetic correlated with activation of TNF signaling and low XIAP expression [6]. Therefore, SMAC mimetics may be particularly useful in cancers associated with high inflammation and NFĸB activation.

Myeloproliferative neoplasm (MPN) is a chronic hematologic malignancy characterized by chronic inflammation [7–9], high TNFα levels in serum [10], activation of NFĸB [11] and reduced expression of XIAP [12]. MPN is characterized by the somatic acquisition of a mutation in either *JAK2* (*JAK2*^*V617F*^) [13–17] or calreticulin (*CALR*) [18, 19] in a hematopoietic stem cell. This mutant clone expands, producing excessive numbers of mature myeloid cells that also carry the driver mutation. The current goals of therapy in MPN are reduction of thrombotic risk, reduction in spleen volume, and alleviation of symptoms. There are no pharmacologic therapies currently in use apart from interferon-alpha that specifically deplete the neoplastic clone and lead to molecular remission [20]. Thus, there is an unmet need to identify therapeutics to successfully target *JAK2*^*V617F*^ cells in MPN. The SMAC mimetic LCL-161 is currently in a Phase 2 clinical trials for the MPN subtype myelofibrosis (NCT02098161) [21]. However, it still remains unclear how LCL-161 impacts *JAK2*^*V617F*^ or *CALR* mutant cells compared to wildtype counterparts.

Based upon the fact that there is hyperactivation of NFĸB signaling pathway [22] we reasoned that MPN cells would be more susceptible to killing by SMAC mimetics. To test this, we performed cell viability assays using murine and human cell lines, myeloid colony formation assays using primary MPN patient cells and a mouse model to test the effect of the SMAC mimetic LCL-161 on *JAK2*^*V617F*^ cells in the presence and absence of TNFα. Here, we find that murine and human *JAK2*^*V617F*^ cell lines are hypersensitive to the SMAC mimetic LCL-161 in vitro in the absence but not presence of TNFα. Furthermore, LCL-161 reduced myeloid colony formation to a greater extent in MPN mice and patients and reduced spleen size and possibly myelofibrosis in vivo.

## Methods

### Drug formulation

LCL-161 was provided by Novartis Pharmaceutical Corporation. For in vitro assays, LCL-161 was dissolved in DMSO at a concentration of 10 mM, stored in single use aliquots at − 80 °C, and dissolved directly in culture media fresh for each experiment. LCL-161 was formulated for oral gavage by dissolving in 0.1 N HCl to a final concentration of 10 mg/ml in sodium acetate buffer (100 mM, pH 4.6). LCL-161 was prepared by first wetting 10 mg powder stock with 30 μl water, and then dissolving in two equivalents (0.73 μl/mg of compound) of 6.0 N HCl. The resulting (clear) solution was brought up to 1 ml in pH 4.6 acetate buffer, and the resulting stock was stored frozen at − 20 °C until used for gavage treatment of mice.

### Creation of L929 cell lines

Parental L929 cell line (ATCC^®^ CCL-1™, purchased from ATCC) were infected with retrovirus containing JAK2^WT^, JAK2^V617F^, CALR^DEL^, CALR^WT^, MPL, or empty vector containing green fluorescent protein (GFP) or human CD4 (hCD4) tags by spinoculation. GFP+ and/or hCD4+ cells were sorted on an Aria flow cytometer (BD) and expanded in culture.

### Protein extraction and Western Blot

Following washes with PBS, cells were lysed in 1× Immunoprecipitation buffer (20 mM Tris–HCl pH 7.5, 150 mM NaCl, 1 mM EDTA pH 8.0, 1 mM EGTA, 1% Triton X-100, 10 mM sodium pyrophosphate, 10 mM α-glycerophosphate, 1 mM Na_3_VO_4_, and 50 mM NaF) by vortexing for 10 s and placing cells on ice for 10 min. Next, samples were centrifuged at 12,000×*g* for 15 min at 4° C. Supernatants were collected and 4× Lamelli buffer (Bio-Rad) was added to each sample prior to storage at − 20 °C. Protein concentration was determined by Bicinchoninic acid (BCA) assay at absorbance at 532 nm. Western blot analysis was performed using 30 µg for protein boiled for 10 min at 95 °C and electrophoresis was carried out on a 10% acrylamide gel. The protein was transferred onto a nitrocellulose membrane and blocked with 5% BSA in 0.1% PBS-T at 25 °C. The membrane was incubated for 1 h at room temperature or overnight at 4 °C with the primary antibodies for cIAP1 (ab2399, Abcam), cIAP2 (ab23423, Abcam), XIAP (ab28151, Abcam), and β-actin (ab8227, Abcam). Following three 15 min wash steps with 0.1% PBS-T, membranes were incubated with secondary antibody goat-anti-rabbit IgG for 1 h. Following an additional wash step, membranes were imaged on a Chemdoc luminescence reader (Bio-Rad).

### Human cell lines and apoptosis assays

K562 and HEL cells (gift from Brian Druker Lab, Oregon Health & Science University) were maintained in RPMI 1640 (Corning) containing phenol red supplemented 100 U penicillin, 100 µg/ml streptomycin, 2 mM glutamine, and 10% FBS. Cells were cultured in a humidified tissue culture incubator at 37 °C and 5% CO_2_. For apoptosis assays, K562 and HEL cells were plated into 12-well plates at a density of 1 × 10^6^ cells per well in RPMI+ 10% FBS + Pen/Strep/l-glutamine containing vehicle, LCL-161, or LCL-161 + TNFα. Following 16 h, flow cytometry for annexin-V and propidium iodide (PI) was performed.

### Murine colony formation assays

Bone marrow was harvested from JAK2^V617F^ knock-in mouse model of MPN and cells were plated at a density of 10,000 cells/ml in Methocult M3230 (stem cell) supplemented 10 ng/ml mouse interleukin-3 (mIL-3), 3 U/ml human erythropoietin (hEPO), and 50 ng/ml mouse stem cells factor (mSCF), all purchased from Peprotech. The cells were plated in triplicate at a density of 10,000 cells/ml and incubated at 37 °C in a 5% carbon dioxide humidified incubator for 5–7 days. Standard morphological criteria were used to score hematopoietic colonies using a light microscope.

### Collection, isolation and culture of primary human samples

All participants gave their informed consent for the studies conducted in accordance with the Declaration of Helsinki. This study was approved by the Institutional Review Board of the University of California, Irvine (IRB HS #2014-9995). Peripheral blood samples were collected from MPN patients with Polycythemia Vera, Essential Thrombocytopenia, and Primary Myelofibrosis or normal controls. Density gradient centrifugation with Ficoll was performed to isolate blood mononuclear cells (PBMCs). Following PBMC isolation, a red blood cell lysis step was performed with ammonium chloride buffer (ACK).

### Human hematopoietic colony assays

Isolated PBMCs were plated in Methocult H4320 (Stem Cell Technologies, Vancouver Canada) supplemented 10 ng/ml human interleukin-3 (hIL-3), 3 U/ml human erythropoietin (hEPO), and 50 ng/ml human stem cells factor (hSCF). The cells were plated in triplicate at a density of 100,000 cells/ml and incubated at 37 °C in a 5% CO_2_ humidified incubator for 7–10 days. Standard morphological criteria were used to score hematopoietic colonies using a light microscope.

### Mice

All mouse work was performed with approval from the IACUC committee at University of California, Irvine. Equal numbers of male and female mice were used for all experiments. The Jak2^V617F^ was a gift from Ann Mullally, Brigham and Women’s Hospital. The transduction–transplantation mouse model of JAK2^V617F^ driven-MPN was established as previously described [10, 23].

### Statistics

All statistical analyses were performed using Graphpad Prism version 7.0 (Graphpad, La Jolla, CA, USA). Statistical analyses were performed using an unpaired t-test (Holm–Sidak) or 2way ANOVA (Tukey’s test). Significant differences were indicated as *P < 0.05, **P < 0.01, ***P < 0.001, ***P < 0.0001. Error bars represent mean values ± SEM. All experiments were performed at least three independent times unless otherwise stated.

## Results

### JAK2^V617F^ mutant cells are hyper-sensitive to the SMAC mimetic LCL-161 in the absence but not presence of TNFα

**Fig. 1:**
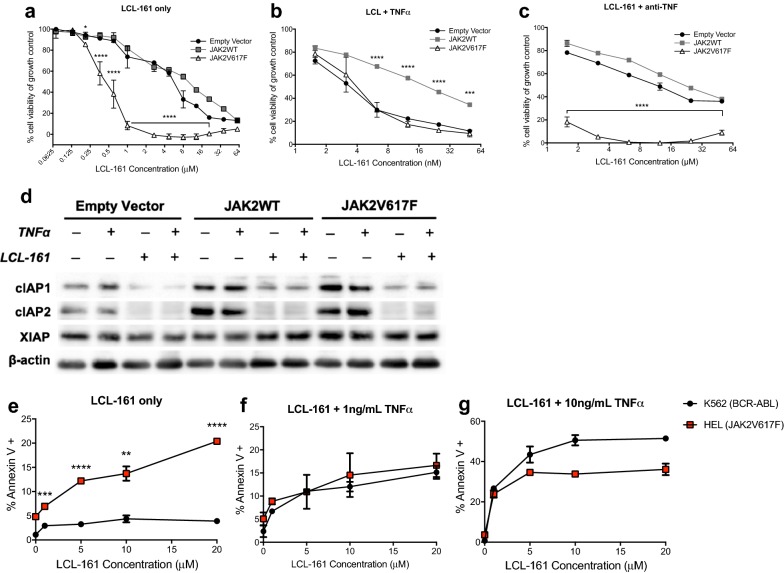
JAK2^V617F^ mutant cells lines are more sensitive to killing by LCL-161 under certain circumstances. **a**–**c**. L929 cells expressing JAK2^WT^, JAK2^V617F^ or empty vector were incubated with increasing concentrations of **a** LCL-161 alone, **b** LCL-161 with the addition of 0.25 ng/ml mTNFα, **c** LCL-161 with the addition of 400 ng/ml mTNFa neutralizing antibody for 48 h and then analyzed with a resazurin based viability assay. ****P < 0.0001, 2way ANOVA. **d** Western blot of L929 cell lines harvested 24 h after exposure to combinations of LCL-161 and mTNFα. **e**–**g** HEL and K562 cells were incubated with **e** LCL-161 alone, **f** LCL-161 + 1 ng/ml hTNFα, or **g** LCL-161 + 10 ng/ml hTNFα for 48 h. Apoptosis was measured with Annexin V and PI staining. **P < 0.01, ****P < 0.0001 unpaired t test

To investigate the sensitivity of JAK2^V617F^ mutant cells to LCL-161, we created cell lines with stable expression of JAK2^V617F^, JAK2^WT^, or empty GFP vector by retroviral transduction. In this assay, we utilized the mouse fibroblast L929 line which are extremely sensitive to TNFα-induced death. We measured the impact of increasing concentrations of LCL-161 on the viability of these cells using a resazurin based assay in the presence or absence of TNFα (Fig. 1a–c). Sub-micromolar concentrations of LCL-161 significantly reduced the viability of L929 JAK2^V617F^ cells compared to JAK2^WT^ or empty vector cells (Fig. 1a). We observed 50% cell viability of L929 JAK2^V617F^ cells with 0.45 µM LCL-161 as compared to similar cell viabilities with 5.5 µM and 9.5 µM LCL-161 for empty vector or JAK2^WT^ cells, respectively (*P *< 0.0001, 2way ANOVA; Fig. 1a). However, this differential sensitivity was lost when TNFα was added to culture (Fig. 1b). Interestingly, overexpression of JAK2^WT^ rendered L929 cells resistant to killing by higher concentrations of LCL-161 in the presence of TNFα, as these cells were 50% viable in the presence of 19 µM LCL-161 (*P *< 0.0001, 2way ANOVA; Fig. 1b).

Next, we assessed whether autonomous production of TNFα could explain the increased sensitivity of JAK2^V617F^ cells to LCL-161. To do this, we tested whether TNFα neutralizing antibody abrogates the increased sensitivity of JAK2^V617F^ mutant L929 cells to LCL-161. Addition of TNFα neutralizing antibody did not rescue the increased sensitivity of JAK2^V617F^ cells to LCL-161 (Fig. 1c) suggesting that the sensitivity of JAK2^V617F^ cells was not due to autonomous production of TNFα. Moreover, we could not detect any TNFα in the supernatant of L929 JAK2^V617F^ cells, either at baseline or with the addition of LCL-161 by ELISA (data not shown). As expected, treatment with LCL-161 decreased both cIAP1 and cIAP2 (Fig. 1d). We found no marked differences in expression of cIAP1, cIAP2, or XIAP in JAK2^V617F^ versus JAK2^WT^ or parental L929 cells under any conditions tested (Fig. 1d). However, we did find evidence that sensitivity to LCL-161 is mediated at least in part to NFĸB activation, the NFĸB inhibitor QNZ had a protective effect on empty vector cells in the presence but not absence of TNF, whereas QNZ had a protective effect on JAK2^V617F^ mutant cells only in the absence of TNF (Additional file 1: Figure S1). This suggests that JAK2^V617F^ induces NFĸB activation in L929 cells and this mediates the sensitivity to LCL-161.

We also compared the ability of LCL-161 to induce apoptosis in the HEL human erythroleukemia cell line which harbors the *JAK2*^*V617F*^ mutation versus the K562 human erythroleukemia cell line which harbors the *BCR*-*ABL* translocation. LCL-161 induced apoptosis significantly more in HEL cells as compared to K562 cells in the absence (*P *< 0.001, unpaired t test; Fig. 1e) but not presence of TNFα (Fig. 1f, g). Likewise, expression levels of cIAP1, cIAP2, or XIAP did not correlate with sensitivity to LCL-161 in HEL human cells expressing JAK2^V617F^ as compared to K562 cells (Additional file 1: Figure S2). These data in human cell lines corroborate our findings in L929 cells that JAK2^V617F^ mutant cells are more sensitive to killing by LCL-161 alone, but when TNFα is added this differential sensitivity is lost.

### Calreticulin mutated cells are not hyper-sensitive to LCL-161

To investigate the effect of LCL-161 on calreticulin mutant cells, we also created L929 cell ectopically expressing MPN associated calreticulin (CALR) mutations with and without its obligate cytokine receptor scaffold thrombopoietin receptor (MPL). CALR mutant L929 cells were not more sensitive to LCL-161 as compared to empty vector either with or without co-expression of MPL (Additional file 1: Figure S3). Interestingly, overexpression of wild-type CALR led to increased sensitivity to LCL-161 when co-expressed with MPL as compared to cells with empty vector, CALR deletion or CALR insertion (Additional file 1: Figure S3).

### Sensitivity of JAK2^V617F^ cells to LCL-161 is dependent upon Janus kinase activity

**Fig. 2:**
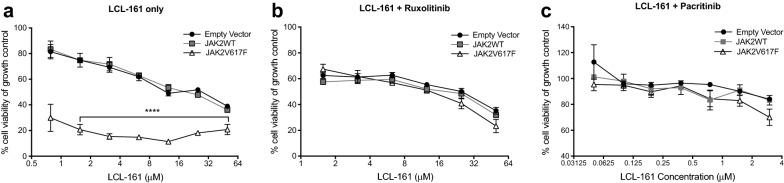
JAK inhibitors rescue hypersensitivity of JAK2^V617F^ mutant cells to LCL-161. L929 cells expressing JAK2^WT^, JAK2^V617F^ or empty vector were incubated with increasing concentrations of **a** LCL-161 alone, **b** LCL-161 with the addition of 1 µM ruxolitinib, or **c** LCL-161 with the addition of 1 µM pacritinib. After 48 h in culture a resazurin-based cell viability assay was performed. ****P < 0.0001, 2way ANOVA

To determine whether the increased sensitivity of JAK2^V617F^ cells to LCL-161 is dependent upon Janus kinase (JAK) activity, we utilized the JAK inhibitors ruxolitinib (JAK1/2 inhibitor) and pacritinib (JAK2/Flt3 inhibitor) in a resazurin based cell viability assay. Sensitivity of JAK2^V617F^ L929 cells was dependent upon activation of JAK2 activity, as treatment with either ruxolitinib or pacritinib reversed the increased sensitivity of JAK2^V617F^ cells to LCL-161 (Fig. 2). This data suggests that the hyper-sensitivity of JAK2^V617F^ cells to LCL-161 is due to constitutive JAK2 activation.

### LCL-161 reduces myeloid colony formation in JAK2^V617F^ knock-in mice and MPN patient cells in the absence of TNFα

**Fig. 3:**
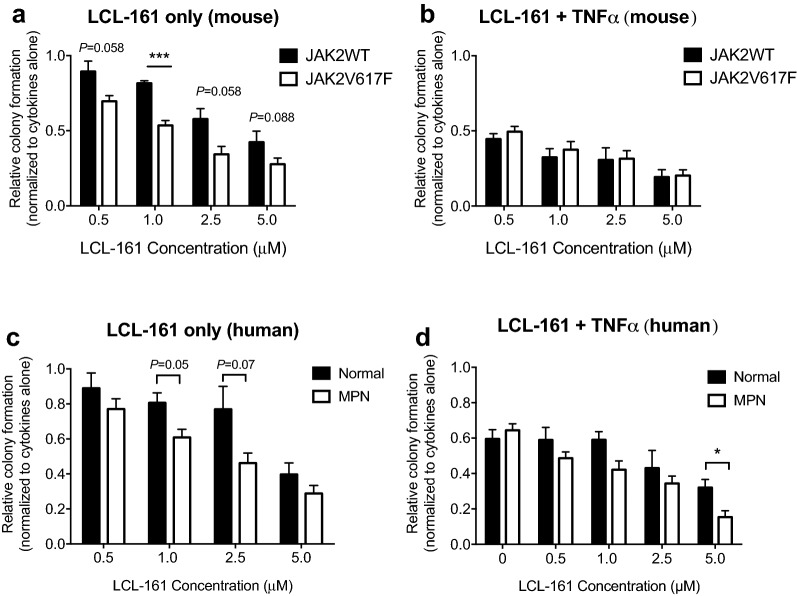
LCL-161 preferentially decreases MPN colony formation. Methylcellulose colony formation of **a**, **b** whole bone marrow from JAK2^V617F^ or wild-type mice and **c**, **d** peripheral blood mononuclear cells from *JAK2*^*V617F*^ mutated MPN patients or normal controls with increasing concentrations of LCL-161 alone (**a**, **c**) or LCL-161 + 0.25 ng/ml TNFα (**b**, **d**). *P < 0.05, ***P < 0.001, unpaired t test

To investigate the effect of LCL-161 on myeloid colony formation in primary mouse bone marrow cells, we performed colony formation assays from JAK2^V617F^ knock-in and wild-type mice with increasing concentrations of LCL-161. LCL-161 reduced colony formation in JAK2^V617F^ cells compared to wild-type cells in the presence of all doses tested, although only the 1 µM LCL-161 dose reached statistical significance (P < 0.001, unpaired t-test; Fig. 3a). However, the differential sensitivity of JAK2^V617F^ cells was lost in the presence of TNFα (Fig. 3b). This data further supports the model that JAK2^V617F^ cells have increased sensitivity to SMAC mimetics in the absence but not presence of TNFα.

We next investigated the effect of LCL-161 on colony formation using primary cells from MPN patients compared to unaffected individuals. In the presence of LCL-161 alone, there was a reduction in colony formation from *JAK2*^*V617F*^ mutated MPN patients versus normal controls at 1 µM and 2.5 µM doses, though this trend did not reach significance (*P *= 0.05 and 0.07, respectively, unpaired t test; Fig. 3c). These same trends were observed when erythroid and G/M colonies were enumerated separately (Additional file 1: Figure S4). When TNFα was added to the culture, this diminished the apparent difference between MPN and normal controls in response to LCL-161 at all but the highest concentration (5 µM) of LCL-161 (*P *< 0.05, unpaired t test; Fig. 3d).

### LCL-161 reduces spleen size and ameliorates fibrosis in a JAK2^V617F^ transduction–transplantation model

**Fig. 4:**
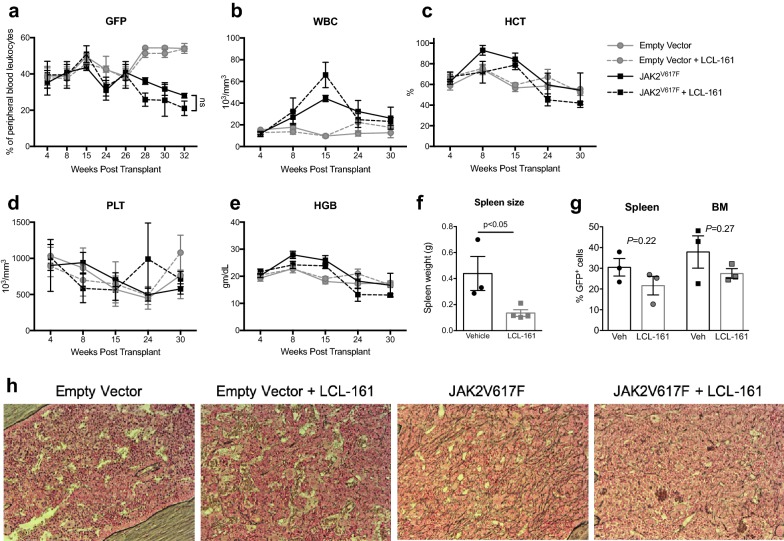
Impact of LCL-161 treatment in a transduction–transplantation model of JAK2V617F mutated MPN. **a** Percentage of GFP+ (empty vector or JAK2^V617F^) cells in peripheral blood. **b** White blood cell (WBC), **c** hematocrit (HCT), **d** platelet (PLT) and **e** hemoglobin (HGB) counts in wild-type (empty vector) or JAK2^V617F^ mice treated with LCL-161. **f** Spleen weights in JAK2^V617F^ mice, *P *< 0.05, unpaired t test. **g** Percentage of GFP+ (JAK2^V617F^) cells in the spleen and bone marrow. **h** Snook’s reticulin staining of paraffin-embedded bone marrows in wildtype (empty vector) and JAK2^V617F^ mice treated with LCL-161. n = 3–4 mice per group

To test the efficacy of LCL-161 in vivo, we utilized a JAK2^V617F^ transduction–transplantation MPN mouse model that develops myelofibrosis around 1 year of age [23–26]. Treatment with LCL-161 was initiated 15 weeks after transplant with twice weekly oral administration. We did not detect changes in peripheral blood counts nor percentage of GFP+ cells in the peripheral blood in LCL-treated mice compared to untreated mice (n = 3–4 mice/group, Fig. 4a–e). However, at sacrifice we found that LCL-161 treatment significantly reduced spleen size (*P *< 0.05, unpaired t test; Fig. 4f) and modestly reduced the percentage of GFP+ cells in the bone marrow and spleen of JAK2^V617F^ mice (n = 3–4 mice/group, Fig. 4g). Upon analysis of reticulin-stained bone marrows, we observed that fibrosis was reduced in mice that had been treated with LCL-161 (Fig. 4h). These data demonstrate that LCL-161 may have a beneficial effect on an in vivo model of JAK2^V617F^ mutated MPN.

## Discussion

Here, we found that mouse cell lines ectopically expressing JAK2^V617F^, the *JAK2*^*V617F*^ mutated human cell line HEL, JAK2^V617F^ knock-in mice, and primary MPN samples were more sensitive to killing by the SMAC mimetic LCL-161 compared to their JAK2^WT^ counterparts. Although addition of exogenous TNFα increased the sensitivity of both mutant and wild-type cells to LCL-161, the differential sensitivity of mutant cells was abolished when TNFα was added to culture. The increased sensitivity to LCL-161 was not due to autocrine production of TNFα, as pharmacologic blockade of TNFα did not rescue JAK2^V617F^ mutant cells from LCL-161.

Activation of JAK2 signaling is critical for the increased sensitivity of JAK2^V617F^ mutant cells to LCL-161. Addition of JAK2 inhibitors ruxolitinib or pacritinib restored LCL-161 sensitivity of JAK2^V617F^ mutant to that of wild-type cells. This observation suggests JAK2 inhibitors may reduce the vulnerability of JAK2 mutant cells to be preferentially killed by SMAC mimetics and that therapeutic combinations of JAK2 inhibitors and SMAC mimetic in patients may not be an ideal combination. This is an important point given that it is common for new investigational agents in myelofibrosis to be tested in combination with ruxolitinib.

The ex vivo experiments with mouse and primary human myeloid colony formation corroborate our findings in cell lines that MPN mutant cells are more sensitive than wild-type cells in the absence but not presence of TNFα. Human MPN patients have variable frequencies of JAK2^V617F^ and so our methylcellulose colony formation assays with primary MPN patient cells assess both mutant and wild-type colonies. This may explain why the impact of LCL-161 on MPN patient colony formation was less robust than our observations in cell lines bearing the JAK2^V617F^ mutation.

Cells bearing the MPN associated CALR^DEL^ mutation were not more sensitive to LCL-161 when expressed either with or without its obligate cytokine receptor scaffold MPL. This suggests that SMAC mimetics may be most beneficial in JAK2^V617F^ mutated MPN patients. CALR binds to and specifically activates MPL, whereas JAK2^V617F^ activates signaling of multiple cytokine receptor pathways. The sensitivity of JAK2^V617F^ to SMAC mimetics is likely mediated via activation of pathways distinct from MPL. Therefore, combination therapy with SMAC mimetic and drugs which target MPL signaling may be additive or even synergistic in JAK2^V617F^ mutated MPN.

In our in vivo mouse model, we found that LCL-161 had modest effects on peripheral blood counts. Although LCL-161 did reduce the percentage of GFP+ cells in the spleen and bone marrow, these effects were modest and did not reach significance. The primary benefit of LCL-161 in this mouse model was reduction in splenomegaly and possible reduction of fibrosis. Future studies would require increasing numbers of mice to validate the effect of LCL-161 on splenomegaly and myelofibrosis in vivo. Regardless, our findings suggest that LCL-161 may not be an ideal single agent in MPN but may be most useful as an adjunct to other MPN therapies.

Approaches that reduce TNFα but maintain constitutive activation of JAK2 in mutant cells may be an ideal combination with SMAC mimetics in MPN. The TNFα decoy receptor etanercept has been investigated as a single agent in an open label pilot study in myelofibrosis and was found to alleviate symptoms [27]. Another potential approach as a combinatorial study with SMAC mimetics would be to combine with a JAK inhibitor that is devoid of JAK2 inhibitory activity, such as itacitinib (INCB039110). This would result in reduction of inflammatory cytokines including TNFα while preserving the vulnerability of JAK2^V617F^ cells to killing by SMAC mimetic.

## Conclusion

SMAC mimetics may be useful in patients with JAK2^V617F^ mutated MPN, as JAK2^V617F^ mutant cells are more readily killed by SMAC mimetics as compared to wild-type cells. This vulnerability to SMAC mimetics is mediated via JAK2 activation, therefore addition of a JAK2 inhibitor to SMAC mimetics may detract from the ability of SMAC mimetics to preferentially kill *JAK2*^*V617F*^ mutant cells. However, therapy with agents that reduce or block TNFα may augment the ability of SMAC mimetics to specifically target *JAK2*^*V617F*^ mutant cells.

## Supplementary information

**Additional file 1: Figure S1.** NFĸB inhibitor protects JAK2V617F mutant cells from killing by LCL-161 in the absence of TNFα and empty vector cells from killing by LCL-161 in the presence of TNFα. L929 cell transduced with empty vector or JAK2^V617F^ were exposed to LCL-161, either in the presence or absence of 1 ng/ml hTNFα and 1nM QNZ. The concentration of LCL-161 used without TNFα was 1.5 µM and with TNFα was 12.5 nM. After 48 h of culture a resazurin based cell viability assay was performed.

**Additional file 2: Figure S2.** Expression levels of cIAP1/2 or XIAP do not influence sensitivity of human cell lines to LCL-161. Western blot of K562 (BCR-ABL mutant) and HEL (JAK2^V617F^) cells treated with 10 nM LCL-161 for 2 h before protein isolation. LCL-161 reduces expression of cIAP1 and cIAP2 in both cell lines as expected. XIAP expression was unaffected by LCL-161.

**Additional file 3: Figure S3.** Calreticulin-mutant cells are not hypersensitive to LCL-161. (A, B) Resazurin-based cell viability assay showing L929 cells transduced with the Calreticulin (CALR) mutations representing empty vector (EV), wild-type CALR (CALR^WT^), deletion (CALR^DEL^) or insertion (CALR^INS^) (A) containing thrombopoietin receptor (MPL) and (B) without MPL treated with increasing concentrations of LCL-161 for 48 h. **P < 0.01, ***P < 0.001 2way ANOVA. (C) Western blot for cIAP1/2, XIAP, and β-Actin as a loading control in CALR^WT^, CALR^DEL^, CALR^INS^, or empty vector (VEH) cells in the presence or absence of MPL. (D) Myeloid colony formation using MNCs from normal controls (n = 5), CALR-mutated patients (n = 5), and JAK2^V617F^ patients (n = 5). Cells were plated in methylcellulose with varying LCL-161 concentrations. Colonies were counted from each plate and normalized to 0 µM LCL-161. Error bar represent mean values ± SEM.

**Additional file 4: Figure S4.** Colony formation shown in Fig. 3 separated by erythroid and G/M colonies. (A) Erythroid and (B) G/M colony formation from MPN patients and normal controls with increasing concentrations of LCL-161. (C) Erythroid and (D) G/M colony formation from MPN patients and normal controls with 10 ng/ml TNFα + increasing concentrations of LCL-161.

## Data Availability

The datasets used and/or analyzed during the current study are available from the corresponding author on reasonable request.

## Abbreviations

BCA: bicinchoninic acid assay
BSA: bovine serum albumin
CALR: calreticulin
EPO: erythropoietin
FBS: fetal bovine serum
GFP: green fluorescent protein
G/M: granulocyte/monocyte
IAP: inhibitor of apoptosis proteins
IL-3: interleukin-3
JAK: Janus kinase
MPL: thrombopoietin receptor
MPN: myeloproliferative neoplasm
NFKB: nuclear factor kappa-light-chain-enhancer of activated B cells
PI: propidium iodide
PBMNC: peripheral blood mononuclear cells
SCF: stem cell factor
SMAC: second mitochondria-derived activator of caspases
TNFα: tumor necrosis factor-alpha
PBS: phosphate buffered saline

## References

[1] Owens TW, Gilmore AP, Streuli CH, Foster FM (2013). Inhibitor of apoptosis proteins: promising targets for cancer therapy. J Carcinog Mutagen.

[2] Chen DJ, Huerta S (2009). Smac mimetics as new cancer therapeutics. Anticancer Drugs.

[3] Fulda S (2015). Smac mimetics as IAP antagonists. Semin Cell Dev Biol.

[4] Welsh K, Milutinovic S, Ardecky RJ (2016). Characterization of potent SMAC mimetics that sensitize cancer cells to TNF family-induced apoptosis. PLoS ONE.

[5] Berger R, Jennewein C, Marschall V (2011). NF-kappaB is required for Smac mimetic-mediated sensitization of glioblastoma cells for gamma-irradiation-induced apoptosis. Mol Cancer Ther.

[6] Lueck SC, Russ AC, Botzenhardt U (2016). Smac mimetic induces cell death in a large proportion of primary acute myeloid leukemia samples, which correlates with defined molecular markers. Oncotarget.

[7] Mondet J, Hussein K, Mossuz P (2015). Circulating cytokine levels as markers of inflammation in philadelphia negative myeloproliferative neoplasms: diagnostic and prognostic interest. Mediat Inflamm.

[8] Hermouet S, Bigot-Corbel E, Gardie B (2015). Pathogenesis of myeloproliferative neoplasms: role and mechanisms of chronic inflammation. Mediat Inflamm.

[9] Fleischman AG (2015). Inflammation as a driver of clonal evolution in myeloproliferative neoplasm. Mediat Inflamm.

[10] Fleischman AG, Aichberger KJ, Luty SB (2011). TNFalpha facilitates clonal expansion of JAK2V617F positive cells in myeloproliferative neoplasms. Blood.

[11] Fisher DAC, Malkova O, Engle EK (2017). Mass cytometry analysis reveals hyperactive NF Kappa B signaling in myelofibrosis and secondary acute myeloid leukemia. Leukemia.

[12] Heaton WL, Senina AV, Pomicter AD (2018). Autocrine Tnf signaling favors malignant cells in myelofibrosis in a Tnfr2-dependent fashion. Leukemia.

[13] Baxter EJ, Scott LM, Campbell PJ (2005). Acquired mutation of the tyrosine kinase JAK2 in human myeloproliferative disorders. Lancet.

[14] Campbell PJ, Baxter EJ, Beer PA (2006). Mutation of JAK2 in the myeloproliferative disorders: timing, clonality studies, cytogenetic associations, and role in leukemic transformation. Blood.

[15] James C, Ugo V, Le Couedic JP (2005). A unique clonal JAK2 mutation leading to constitutive signalling causes polycythaemia vera. Nature.

[16] Kralovics R, Passamonti F, Buser AS (2005). A gain-of-function mutation of JAK2 in myeloproliferative disorders. N Engl J Med.

[17] Levine RL, Wadleigh M, Cools J (2005). Activating mutation in the tyrosine kinase JAK2 in polycythemia vera, essential thrombocythemia, and myeloid metaplasia with myelofibrosis. Cancer Cell.

[18] Klampfl T, Gisslinger H, Harutyunyan AS (2013). Somatic mutations of calreticulin in myeloproliferative neoplasms. N Engl J Med.

[19] Nangalia J, Massie CE, Baxter EJ (2013). Somatic CALR mutations in myeloproliferative neoplasms with nonmutated JAK2. N Engl J Med.

[20] Silver RT, Barel AC, Lascu E (2017). The effect of initial molecular profile on response to recombinant interferon-alpha (rIFNalpha) treatment in early myelofibrosis. Cancer.

[21] Boddu P, Carter BZ, Verstovsek S, Pemmaraju N (2019). SMAC mimetics as potential cancer therapeutics in myeloid malignancies. Br J Haematol.

[22] Fisher DA EE, Malkova O, Fulbright MC, Zhou A, Oh ST. NF Kappa B signaling hyperactivation in myelofibrosis and secondary acute myeloid leukemia. In: American Society of Hematology annual meeting oral abstract. 2015.

[23] Bumm TG, Elsea C, Corbin AS (2006). Characterization of murine JAK2V617F-positive myeloproliferative disease. Cancer Res.

[24] Lacout C, Pisani DF, Tulliez M, Gachelin FM, Vainchenker W, Villeval J-L (2006). JAK2V617F expression in murine hematopoietic cells leads to MPD mimicking human PV with secondary myelofibrosis. Blood.

[25] Zaleskas VM, Krause DS, Lazarides K (2006). Molecular pathogenesis and therapy of polycythemia induced in mice by JAK2 V617F. PLoS ONE.

[26] Wernig G, Mercher T, Okabe R, Levine RL, Lee BH, Gilliland DG (2006). Expression of Jak2V617F causes a polycythemia vera-like disease with associated myelofibrosis in a murine bone marrow transplant model. Blood.

[27] Steensma DP, Mesa RA, Li CY, Gray L, Tefferi A (2002). Etanercept, a soluble tumor necrosis factor receptor, palliates constitutional symptoms in patients with myelofibrosis with myeloid metaplasia: results of a pilot study. Blood.

