# Supplementary figures and images for: The SMAC mimetic LCL-161 selectively targets JAK2V617F mutant cells
Source: Exp Hematol Oncol. 2020 Jan 2;9:1. doi: 10.1186/s40164-019-0157-6 (PMC6941266; doi:10.1186/s40164-019-0157-6)

## LCL +/- QNZ

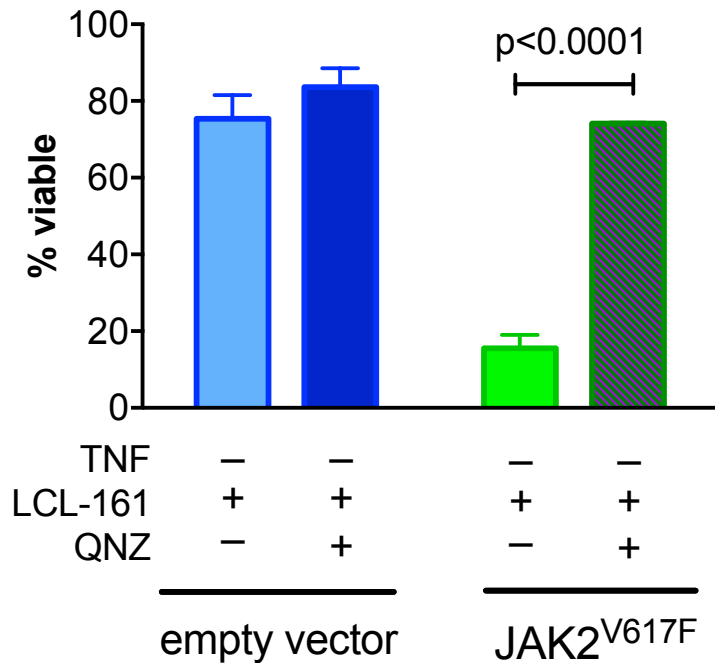

## LCL + TNF +/- QNZ

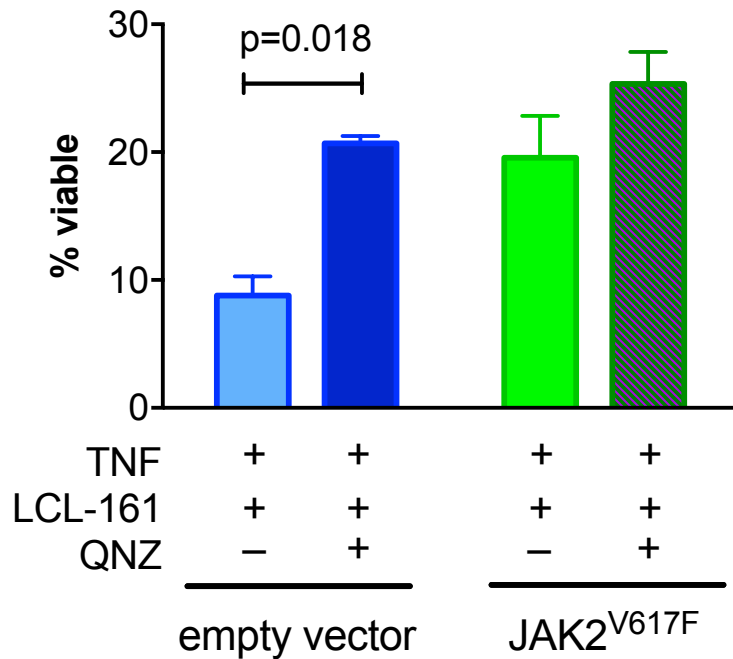

Supplement: Supplementary file 1 — Additional file 1: Figure S1. NFĸB inhibitor protects JAK2V617F mutant cells from killing by LCL-161 in the absence of TNFα and empty vector cells from killing by LCL-161 in the presence of TNFα. L929 cell transduced with empty vector or JAK2V617F were exposed to LCL-161, either in the presence or absence of 1 ng/ml hTNFα and 1nM QNZ. The concentration of LCL-161 used without TNFα was 1.5 µM and with TNFα was 12.5 nM. After 48 h of culture a resazurin based cell viability assay was performed. [file 40164_2019_157_MOESM1_ESM.pdf]

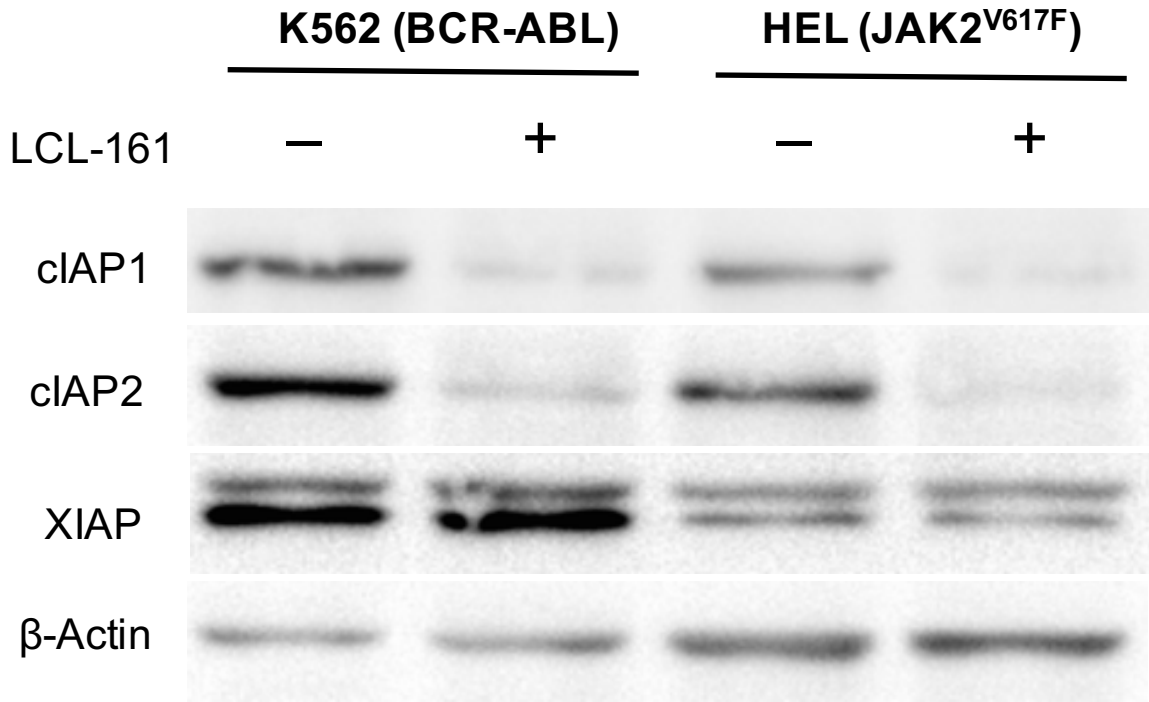

Supplement: Supplementary file 2 — Additional file 2: Figure S2. Expression levels of cIAP1/2 or XIAP do not influence sensitivity of human cell lines to LCL-161. Western blot of K562 (BCR-ABL mutant) and HEL (JAK2V617F) cells treated with 10 nM LCL-161 for 2 h before protein isolation. LCL-161 reduces expression of cIAP1 and cIAP2 in both cell lines as expected. XIAP expression was unaffected by LCL-161. [file 40164_2019_157_MOESM2_ESM.pdf]

**A**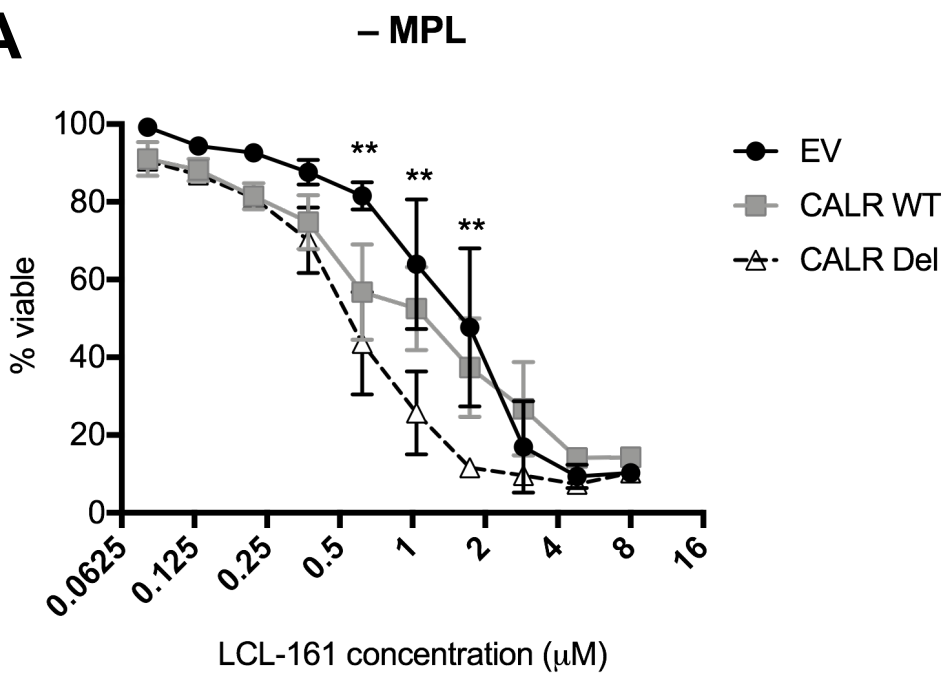**B**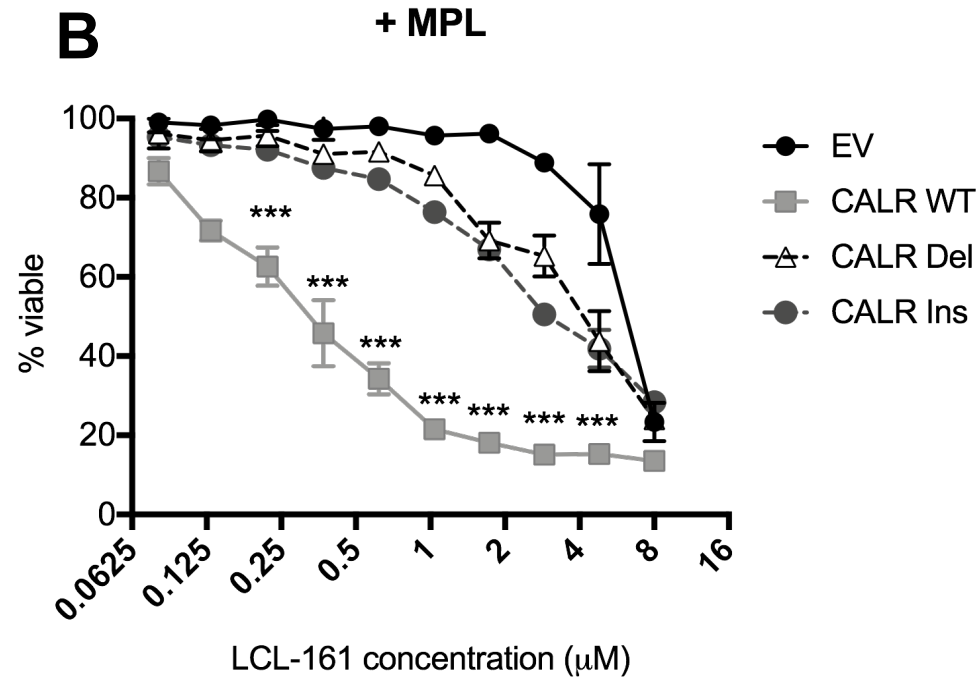**C**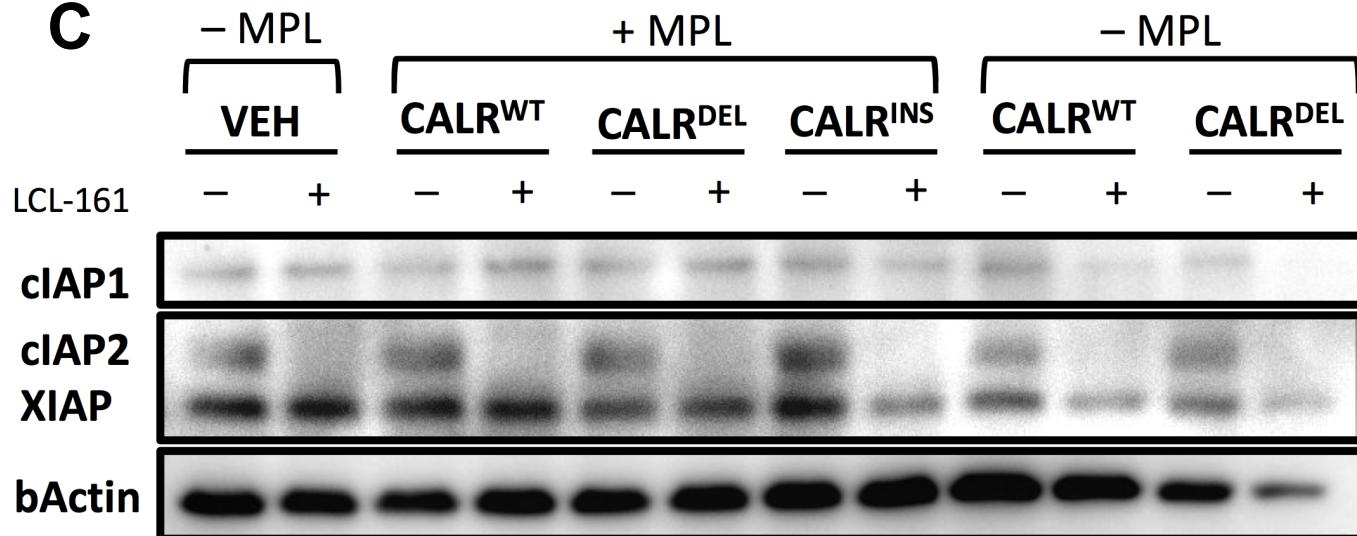**D**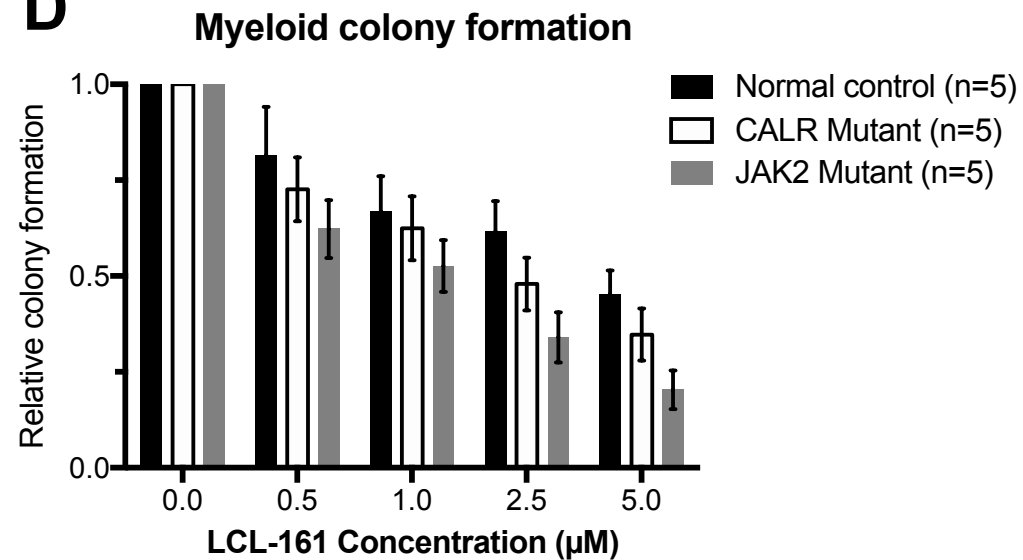

Supplement: Supplementary file 3 — Additional file 3: Figure S3. Calreticulin-mutant cells are not hypersensitive to LCL-161. (A, B) Resazurin-based cell viability assay showing L929 cells transduced with the Calreticulin (CALR) mutations representing empty vector (EV), wild-type CALR (CALRWT), deletion (CALRDEL) or insertion (CALRINS) (A) containing thrombopoietin receptor (MPL) and (B) without MPL treated with increasing concentrations of LCL-161 for 48 h. **P < 0.01, ***P < 0.001 2way ANOVA. (C) Western blot for cIAP1/2, XIAP, and β-Actin as a loading control in CALRWT, CALRDEL, CALRINS, or empty vector (VEH) cells in the presence or absence of MPL. (D) Myeloid colony formation using MNCs from normal controls (n = 5), CALR-mutated patients (n = 5), and JAK2V617F patients (n = 5). Cells were plated in methylcellulose with varying LCL-161 concentrations. Colonies were counted from each plate and normalized to 0 µM LCL-161. Error bar represent mean values ± SEM. [file 40164_2019_157_MOESM3_ESM.pdf]

**A****Erythroid**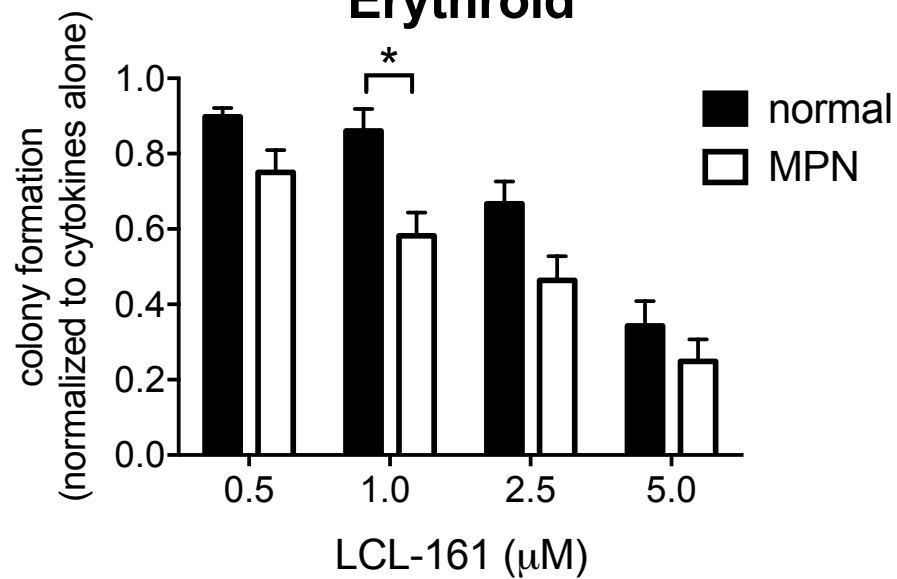**B****GM**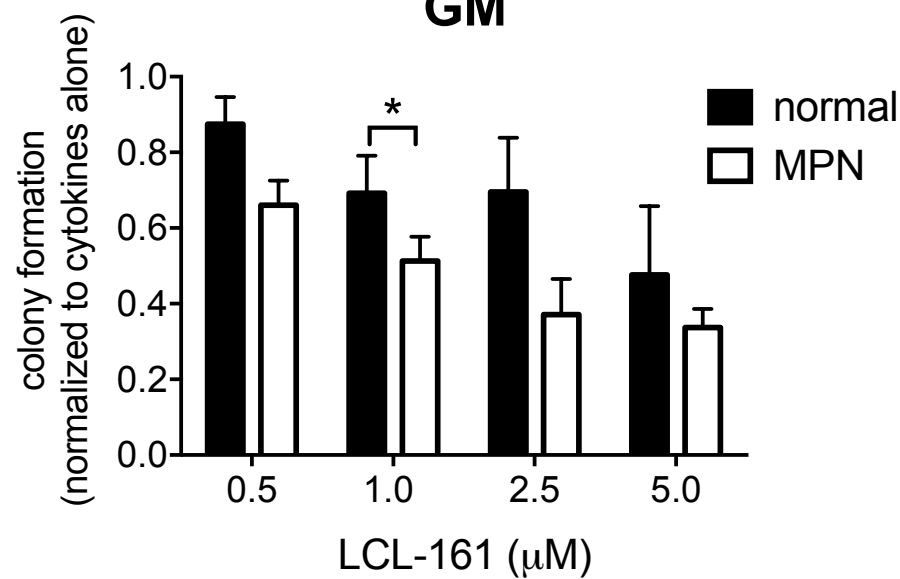**C****Erythroid**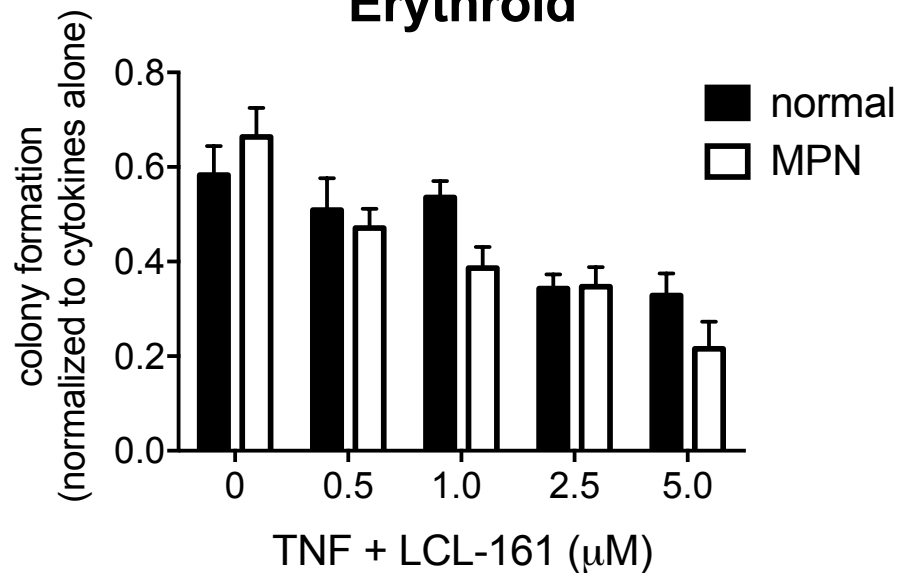**D****GM**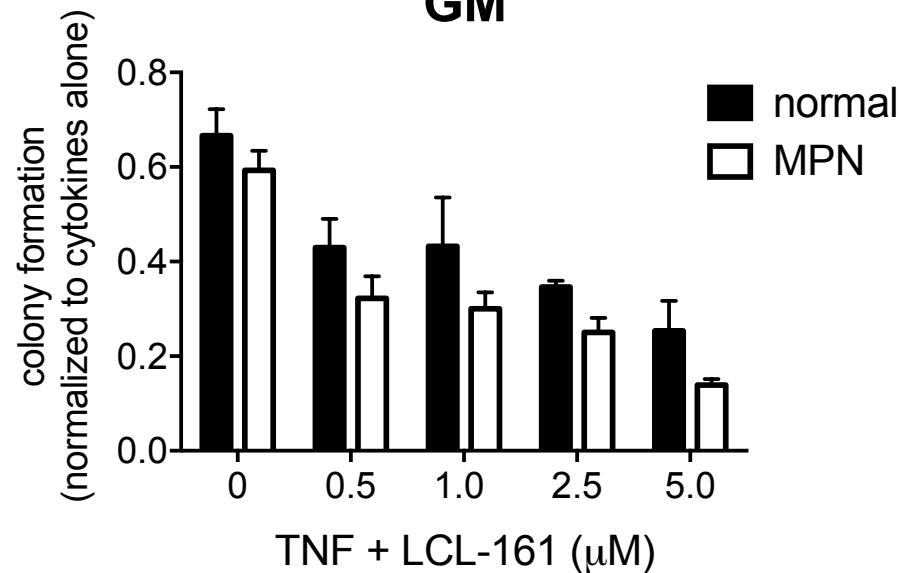

Supplement: Supplementary file 4 — Additional file 4: Figure S4. Colony formation shown in Fig. 3 separated by erythroid and G/M colonies. (A) Erythroid and (B) G/M colony formation from MPN patients and normal controls with increasing concentrations of LCL-161. (C) Erythroid and (D) G/M colony formation from MPN patients and normal controls with 10 ng/ml TNFα + increasing concentrations of LCL-161. [file 40164_2019_157_MOESM4_ESM.pdf]
